# Five near-infrared-emissive graphene quantum dots for multiplex bioimaging

**DOI:** 10.1088/2053-1583/ad1c6e

**Published:** 2024-01-19

**Authors:** Alina R Valimukhametova, Olivia Fannon, Ugur C Topkiran, Abby Dorsky, Olivia Sottile, Roberto Gonzalez-Rodriguez, Jeffery Coffer, Anton V Naumov

**Affiliations:** 1 Department of Physics and Astronomy, Texas Christian University, TCU Box 298840, Fort Worth, TX 76129, United States of America; 2 Department of Physics, University of North Texas, Denton, TX 76203-1277, United States of America; 3 Department of Chemistry and Biochemistry, Texas Christian University, TCU Box 298860, Fort Worth, TX 76129, United States of America

**Keywords:** graphene quantum dots, rare-earth metals, near-infrared fluorescence, fluorescence microscopy

## Abstract

Due to high tissue penetration depth and low autofluorescence backgrounds, near-infrared (NIR) fluorescence imaging has recently become an advantageous diagnostic technique used in a variety of fields. However, most of the NIR fluorophores do not have therapeutic delivery capabilities, exhibit low photostabilities, and raise toxicity concerns. To address these issues, we developed and tested five types of biocompatible graphene quantum dots (GQDs) exhibiting spectrally-separated fluorescence in the NIR range of 928–1053 nm with NIR excitation. Their optical properties in the NIR are attributed to either rare-earth metal dopants (Ho-NGQDs, Yb-NGQDs, Nd-NGQDs) or defect-states (nitrogen doped GQDS (NGQDs), reduced graphene oxides) as verified by Hartree-Fock calculations. Moderate up to 1.34% quantum yields of these GQDs are well-compensated by their remarkable >4 h photostability. At the biocompatible concentrations of up to 0.5–2 mg ml^−1^ GQDs successfully internalize into HEK-293 cells and enable *in vitro* imaging in the visible and NIR. Tested all together in HEK-293 cells five GQD types enable simultaneous multiplex imaging in the NIR-I and NIR-II shown for the first time in this work for GQD platforms. Substantial photostability, spectrally-separated NIR emission, and high biocompatibility of five GQD types developed here suggest their promising potential in multianalyte testing and multiwavelength bioimaging of combination therapies.

## 1. Introduction

Near-infrared (NIR) imaging is one of the most rapidly developing prospective techniques in biomedical diagnostics. It possesses all the attributes of regular visible fluorescence-based diagnostic imaging, while enabling over 10–100 times [1, 2] deeper tissue penetration and detection with lower biological autofluorescence. This occurs within several optical windows of 650–950 nm (NIR-I), 1000–1350 nm (NIR-II), and 1550–1870 nm (NIR-III) suitable for biomedical imaging [3, 4]. Recent studies show that the highest tissue transmission starts at 950 nm [5] and the biological autofluorescence is minimized above 1000 nm [3], while the fluorescence of the FDA-approved indocyanine green (ICG) and a variety of other NIR dyes in the development is generally below those thresholds. Furthermore, NIR dyes possess substantially lower quantum yields (QY) than conventional fluorophores [6, 7]. This disadvantage can be circumvented by having higher fluorophore photostability allowing longer image acquisition times sufficient for autofluorescence photobleaching and accumulation of substantial signal over noise. Fluorophore photostability may also play an important role in the NIR applications requiring multiple hours of patient observation. However, all currently used NIR dyes do not possess sufficient photostability to utilize those advantages. Given the advent of drug delivery, the ability of the biomarkers to aid in drug transport or trace multiple combination therapeutics at the same time to the disease site is also highly desired. All these unmet requirements indicate the critical need in the development of several biocompatible photostable NIR imaging agents capable of multiple drug tracing and delivery. New developments in nanotechnology can aid in undertaking this challenge as fluorescence properties of nanomaterials can often be tailored synthetically to address multiple aforementioned needs.

While conventional fluorescence markers often have higher QYs than nanomaterial platforms, the latter are generally more photostable and, in the long run, can provide effective and quantitative fluorescence tracking and assessment of payload bioavailability. Inorganic nanomaterials, such as single-walled carbon nanotubes (SWCNTs) [8–11], semiconductor quantum dots (QDs) [12, 13], quantum rods [14] and rare-earth metal nanoparticles [15–17] exhibit fluorescence emission in NIR-I—NIR-III regions having both excitation and emission in the NIR, as desired for bioimaging. Due to chirality-dependent fluorescence in the NIR-II, SWCNTs are ideal candidates for multiplex imaging [18]. However, the transition of SWCNTs into clinic is delayed, due to coating-dependent biocompatibility [19], high accumulation/excretion times [20] and insolubility in water without the surfactant. Most of the NIR I/II-emitting semiconductor QDs based on heavy metals [13, 21, 22] and quantum rods synthesized with alloys [14] demonstrate the adverse effects on the environment and possess high biotoxicity. To eliminate toxicity concerns, cadmium-free QDs such as InP [23] and Zn-Cu-In-Se QDs [24] have been developed and utilized for *in vivo* applications. In addition, these semiconductor QDs demonstrate tunable emission with peaks ranging from 515–845 nm for InP QDs, and 630–800 nm for Zn-Cu-In-Se QDs suggesting the possibility for multiplex bioimaging in the NIR-I optical window. Some rare earth-based nanoparticles already demonstrated properties desired for such imaging including photostability within 8 h [25], deep tissue penetration of their emission [15, 16], and substantial QYs of up to 32% [17]. However, their complex core/shell structures and, generally, larger sizes hampering their entry into biological tissues prevent such nanoparticles from direct use in clinics. Finally, the nanomaterials mentioned earlier face limitations due to complicated, expensive or non-eco-friendly synthetic methods, hindering their wide-scale production. Therefore, even though inorganic nanoparticles offer a promising option for NIR imaging and therapeutic transport, there is still a demand for novel nanomaterials with cost-effective, scalable, and eco-friendly synthesis, smaller sizes, NIR emission/excitation capabilities, and high biocompatibility. Ideally, for applications in drug delivery and sensing, these nanomaterials should be easily modifiable though chemical functionalization, allowing the attachment of targeting agents and drug/gene payload [26].

Biocompatible NIR-emissive graphene quantum dots (GQDs) are up to the challenge to satisfy all these preferred conditions. GQDs can be synthesized to exhibit minimal toxicity *in vitro* and *in vivo* due to their small size, water solubility and effective biodegradation or excretion from the cells and organs [27–29]. They can successfully deliver several drug and gene payloads including molecular drugs, plasmids, and nucleic acids [30–32]. While most GQDs fluoresce in the visible, some structures were recently developed to exhibit near-infrared fluorescence arising from electronic states at the functional groups and defect sites [33]. Some of those GQDs emit at shorter NIR wavelengths (650–860 nm) requiring red excitation with less than desirable tissue penetration [34]. Two-photon approaches can circumvent the problem of low wavelength excitation as Kuo *et al* demonstrated multiplex imaging size-sorted GQDs with the 800 nm laser [35]. This technique, however, involves a complex and high-power setup that may not be easily translatable *in vivo*. In our previous works, however, we demonstrated that GQDs ‘top-down’ synthesized from reduced graphene oxide (RGQDs) or doped with ${\text{Tm}}^{3+}$ ions exhibit fluorescence at 950 nm with 808 nm NIR-I excitation, while showing substantial biocompatibility at 1 mg ${\text{ml}}^{-1}$ *in vitro* and *in vivo* [26, 29]. In order to produce a whole range of fluorophores emitting at different NIR wavelengths and enabling multiplex imaging, doping with a variety of rare-earth metal ions can be utilized [29, 36]. For instance, trivalent rare-earth metals, such as ${\text{Nd}}^{3+}$, ${\text{Yb}}^{3+}$, ${\text{Tm}}^{3+}$, ${\text{Ho}}^{3+}$, and ${\text{Er}}^{3+}$, exhibit fluorescence in the NIR-I, -II, -III spectral regions caused by their internal electron transitions [37]. According to our previous work [29], few percent doping with ${\text{Tm}}^{3+}$ and ${\text{Nd}}^{3+}$ does not affect the biocompatibility of the core GQD structures, while already enabling NIR fluorescence imaging. In the present work, we use these previous developments to create five spectrally-separated biocompatible GQD fluorophores and test those for simultaneous multi-wavelength imaging applications. These include previously explored RGQDs and Nd-NGQDs as well as never before synthesized Ho-NGQDs and Yb-NGQDs and nitrogen-doped NGQDs for the first time utilized for NIR internalization imaging. Biocompatibilities, QYs, photostabilities and cellular internalization capabilities of these nanostructures are assessed in the NIR to evaluate their ultimate potential for NIR-I and NIR-II multiplex bioimaging.

## 2. Materials and methods

### 2.1. Synthesis of NGQDs, Nd-NGQDs, Ho-NGQDs, Yb-NGQDs, and RGQDs

NGQDs, Nd-NGQDs, and RGQDs were synthesized following the procedure developed in our previous works [28, 29, 38]. Yb-NGQDs, Ho-NGQDs, and Nd-NGQDs are doped with rare earth metal ions rather than zero-valent metals. In order to produce Ho-NGQDs, Yb-NGQDs, Nd-NGQDs and Er-NGQDs, 0.04 M of glucosamine hydrochloride (346299, Sigma-Aldrich) and 0.008 M of Ho(NO_3_)_3_·5H_2_O (14483–18-2, Thermo Scientific), or 0.008 M of YbCl_3_·6H_2_O (337927, Sigma-Aldrich), or 0.008 M of Nd(NO_3_)_3_·6H_2_O (Sigma-Aldrich), or 0.008 M of Er(NO_3_)_3_·H_2_O (12920, Alfa Aesar) were dispersed in 250 ml of distilled water and were treated for 60 min in 1100W, Proctor Silex, microwave oven (model: PSCMZFG13S211, power level 3). To remove unreacted material, the product was dialyzed using 1 kDa molecular weight cutoff (MWCO) dialysis bags for 24 h against distilled water. The water was changed every 30 min for the first 3 h followed by further changing it every 7 h. The purified material was further filtered through a 0.22 *μ*m syringe filter removing any larger aggregates and sterilizing the sample.

### 2.2. Structural and optical characterization

The size distribution and morphology of NGQDs, Nd-NGQDs, Ho-NGQDs, Yb-NGQDs, and RGQDs was assessed via the HRTEM (high-resolution transmission electron microscopy, JEOL JEM-2100) with energy dispersive x-ray analysis (EDS, JEOL, Peabody, MA, USA). The samples were air-dried on the carbon-coated 200-mesh copper grid. The presence of functional groups on the GQD surface was assessed via the ATR mode of the Thermo Nicolet Nexus 670 FTIR after freeze-drying samples in Labconco FreeZone 4.5 freeze-dryer. The absorbance of NGQDs, Nd-NGQDs, Ho-NGQDs, Yb-NGQDs, and RGQDs was measured within the range of 200–1000 nm with Agilent Technologies (Cary 60 UV–vis) absorption spectrometer. Yb-NGQDs absorbance within the range of 950–1200 nm was measured using NS2 NanoSpectralyzer (Applied NanoFluorescence, Houston, TX, USA). Fluorescence spectra of the samples in the visible and NIR, as well as the QY in the NIR, were measured utilizing Horiba Scientific SPEX NanoLog spectrofluorometer. Due to the proximity of the excitation of the Yb-NGQDs at 980 nm to their emission at 997 nm excitation laser line was subtracted from their spectra.

### 2.3. QY and photostability measurements

A comparative approach was utilized to calculate the QY of GQDs choosing ICG (2.5% QY in water [39]) as a reference material. 808 nm excitation at a power density of 0.08 W cm^−2^ and 650 nm excitation at a power density of 0.2 W cm^−2^ were utilized. The following formula was used to calculate the QY of GQDs:

$${\text{QY}}_{\text{GQDs}}={\text{QY}}_{\text{ref}}\times \left(\frac{{\text{FLI}}_{\text{GQDs}}}{{\text{FLI}}_{\text{ref}}}\right)\times \left(\frac{1-10^{-A_{\text{ref}}}}{1-10^{-A_{\text{GQDs}}}}\right)\times {\left(\frac{n_{\text{GQDs}}}{n_{\text{ref}}}\right)}^{2}.$$

In the above expression, ${\text{QY}}_{\text{ref}}$ denotes the QY of ICG dye, ${\text{FLI}}_{\text{GQDs}}$ and ${\text{FLI}}_{\text{ref}}$ represents experimentally measured integrated fluorescence intensity of GQDs and ICG, and indicates the absorbance of GQDs and ICG at the excitation wavelength. The refractive index of water is a function of the wavelength, therefore, the following refraction indexes of water based on the GQD and ICG maximum emission peaks were used for each sample: $n_{\text{ref}}(871\text{nm})=1.324244$, $n_{\text{Ho-NGQDs}}(967\text{nm})=1.322249$, $n_{\text{NGQDs}}(983\text{nm})=1.321994$, $n_{\text{Nd-NGQDs}}(1053\text{nm})=1.320775$ [40]. Photostability of NGQDs, Nd-NGQDs, RGQDs, ICG, Ho-NGQDs, and Yb-NGQDs in aqueous suspension was measured under continuous 808 nm, 650 nm, and 980 nm laser exposure at a power density of 0.2 W cm^−2^ [25, 41]. A 1 mm pathlength quartz cuvette was utilized for photostability measurements to avoid diffusion of the non-exposed to irradiation GQDs.

### 2.4. Fluorescence microscopy

Fluorescence microscopy measurements were performed using an Olympus IX73 fluorescence microscope with a 60× (IR-corrected Olympus Plan Apo) water immersion objective and Photometrics Prime 95B CMOS camera coupled to Olympus DSU (disk spinning unit) confocal system for visible imaging. Based on visible GQD excitation and emission spectra, the following configuration of filters was chosen: 460 ± 20 nm filter for excitation and 535 ± 20 nm filter for emission [42]. NIR fluorescence was detected by utilizing the NIR InGaAs Photon etc. (ZephIR 1.7) detector through the hyperspectral fluorescence imager Photon etc., enabling spectrally-resolved image acquisition in the near-infrared (850–1600 nm).

### 2.5. Cell studies

HEK-293 cells (CRL-1573, ATCC) were used for cell viability assays and cell internalization studies. Cell viability assay, 3-(4-dimethylthiazol-2-yl)-2,5-diphenyltetrazolium bromide (MTT) assay, was performed to evaluate the biocompatibility of NGQDs, Nd-NGQDs, Ho-NGQDs, Yb-NGQDs, and RGQDs. In short, HEK-293 cells were plated in a 96-well plate at a density of 5000 cells per well and kept in an incubator overnight at 37.1 °C with 5% CO_2_. The next day GQD samples at concentrations of 0.125, 0.25, 0.5, 1, and 2 mg ml^−1^ were added into each well. After 24 h of incubation, the medium was replaced with 100 μl of 1 mg ml^−1^ of MTT. After 4 h of incubation of cells with the MTT, the water-insoluble byproduct, formazan, was dissolved with 100 μl of DMSO. The metabolic activity of living cells was further assessed with absorbance measurements at 580 nm using a FLUOstar Omega microplate reader.

Cell internalization studies were performed with biocompatible 1 mg ml^−1^ concentrations of NGQDs, Yb-NGQDs, Nd-NGQDs, and 0.5 mg ml^−1^ concentrations of RGQDs and Ho-NGQDs. In short, HEK-293 cells were plated onto the coverslips placed in a 6-well plate at a density of 10 000 cells per well. The coverslips have been cleaned and coated with rat tail collagen I (ALX-522–435-0020, Enzo) as per the manufacturer’s protocol for the attachment of HEK-293 cells. The next day all samples were introduced into respective wells and incubated for 1, 6, 12, 24, and 48 h. Coverslips with cells were then washed with 1X phosphate-buffered saline to remove GQDs that had not been internalized. Cells were further fixed between the cover slip and a microscope slide with 4% formaldehyde solution (28 908, Thermo Scientific) and 1× Fluoromount-GTM mounting medium (00–4958-02, Invitrogen). ∼100 cells were imaged at each internalization study time point for each GQD type. Mean fluorescence intensity per unit area was calculated for each cell and deemed proportional to the amount of internalized GQDs.

### 2.6. Computational details

The Ground State of NGQD (C_145_H_35_O_17_N_7_) and RGQD (C_150_H_27_O_16_) monolayer model systems were built using GaussView 6.1 software [43] and optimized employing Ground State Hartree-Fock method [44] at Default Spin with 3–21 G atomic basis set at default temperature of 298.15 Kelvin. The same methodology was utilized for the determination of each models’ energy gaps and fluorescence peaks. All calculations were performed using the Gaussian16 program [45].

## 3. Results and discussion

**Figure 1.**
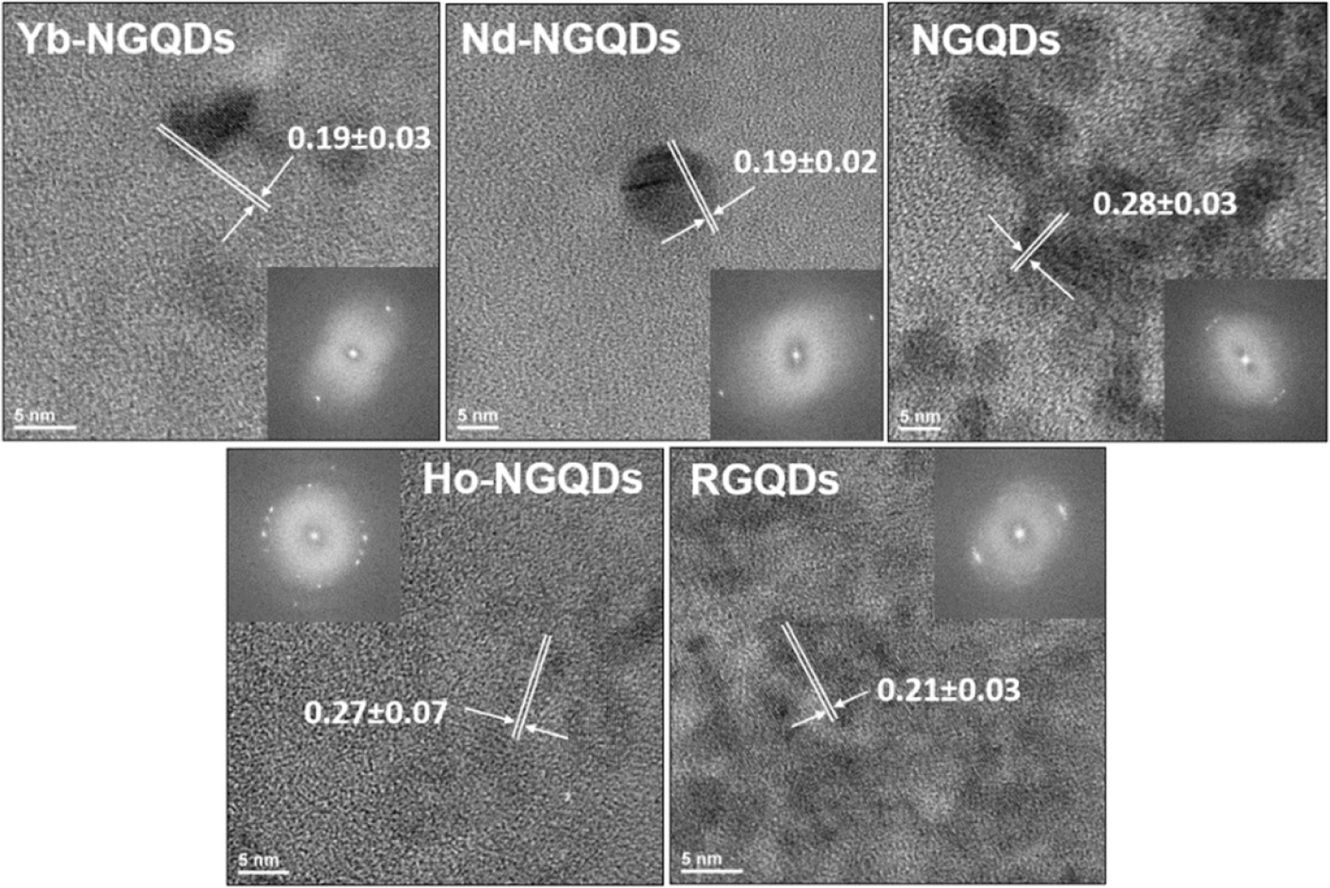
HRTEM images of Yb-NGQDs, Nd-NGQDs, NGQDs, Ho-NGQDs, and RGQDs with corresponding lattice spacing. Insets represent FFT images of the chosen area.

In order to demonstrate the potential of NGQDs, Nd-NGQDs, Ho-NGQDs, Yb-NGQDs, and RGQDs to serve as near-infrared fluorophores in biomedical multiplex imaging applications, we studied their optical properties, cytotoxicity and internalization/excretion in HEK-293 cells. Here, we synthesized NGQDs, Nd-NGQDs, Ho-NGQDs, Yb-NGQDs using a simple ‘bottom-up’ microwave-assisted hydrothermal method with glucosamine serving as a carbon precursor and a nitrogen source [28], while rare-earth metal salts provided ${\text{Nd}}^{3+}$, ${\text{Ho}}^{3+}$, or ${\text{Yb}}^{3+}$ dopants [29]. RGQDs were produced using a ‘top-down’ approach from commercially available reduced graphene oxide, oxidized by sodium hypochlorite photodissociation products [38]. Unreacted precursor materials were removed by 24 h dialysis in 1 kDa dialysis bags against DI water and by filtration through 0.22 *μ*m pore syringe filters, while the latter procedure also served as a sterilization step for all GQD structures. TEM imaging verified the nanoscale sizes of the synthesized NGQDs (5.7 ± 1.0 nm), Nd-NGQDs (5.5 ± 1.1 nm and 11.0 ± 1.6 nm), Ho-NGQDs (5.9 ± 1.2 nm), Yb-NGQDs (5.6 ± 1.8 nm), and RGQDs (4.0 ± 0.7 nm) (figure S1). All the samples showed characteristic ${\text{sp}}^{2}$ graphitic lattice fringes and their spacing within 0.19–0.28 nm range (figure 1), likely corresponding to the (100) and (002) planes of graphene, affected somewhat by defects and heteroatom dopants [46–48]. Concomitant EDX analysis was utilized to verify the doping of GQDs with rare-earth metals (figure S2). Considering that the majority of unreacted precursors were removed by 24 h dialysis, atomic percentages of rare earth metals ranging from 1.01% (Nd) to 2.4% (Ho) reflect their content within the GQDs. The rest of the atomic composition of NGQDs [28], Nd-NGQDs, Ho-NGQDs and Yb-NGQDs consists of carbon, oxygen and nitrogen, while RGQDs contain only carbon and oxygen [38]. Oxygen and nitrogen reside in the form of functional group addends on GQD carbon backbone assessed previously for the core NGQD structures via FTIR [28, 38, 49]. They ensure water solubility of the resulting GQD product critical for biological applications [50, 51].

**Figure 2.**
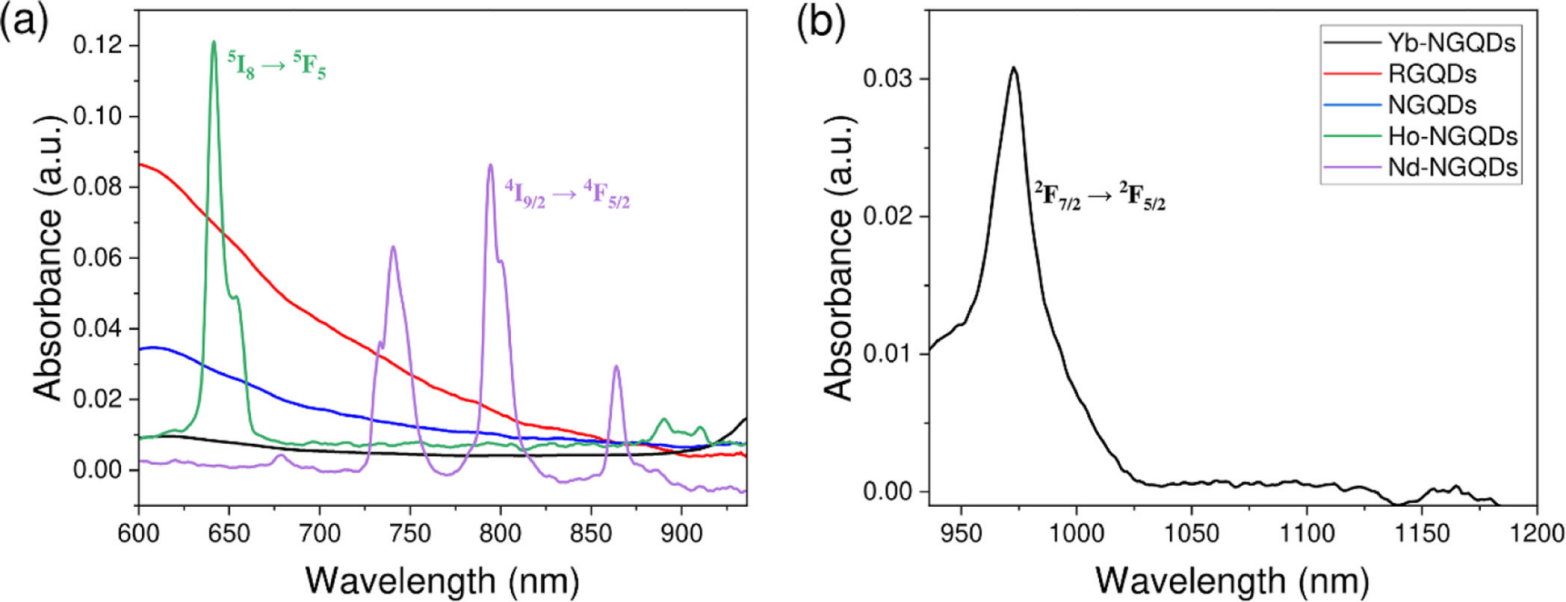
Absorbance spectra of (a) Yb-NGQDs (black), Nd-NGQDs (purple), NGQDs (blue), Ho-NGQDs (green), and RGQDs (red) in the VIS-NIR, and (b) Yb-NGQDs (black) in the NIR.

The optical properties of synthesized GQDs were further assessed via UV–vis and NIR absorbance spectroscopy to evaluate their potential for near-infrared excitation. Similar absorbance peaks corresponding to $\pi -\pi^{*}$ transitions at C=C bounds were observed for both NGQDs and RGQDs at ∼230 nm, while for Nd-NGQDs, Ho-NGQDs, and Yb-NGQDs, this peak appeared to be blue-shifted (figure S3) likely due to the electronic contribution of rare-earth dopants [52]. This further confirms the influence of rare earth metal doping for all three doped nanostructures. All GQDs also exhibit a peak at ∼280 nm, or a shoulder in the case of RGQDs arising from $n-\pi^{*}$ electronic transitions of C=O groups [53]. In addition, NGQDs, Nd-NGQDs, Ho-NGQDs, and Yb-NGQDs demonstrate a characteristic peak at ∼300 nm attributed to $\pi -\pi^{*}$ transitions of C=N groups introduced by glucosamine precursors [53]. VIS-NIR absorbance spectra of NGQDs and RGQDs (figure 2(a)) present a long absorption tail extended beyond the visible end of the spectrum potentially corresponding to the transitions at larger graphitic carbon regions [26]. The NIR absorbance spectra of Ho-NGQDs, Nd-NGQDs (figure 2(a)), and Yb-NGQDs (figure 2(b)) demonstrate characteristic rare-earth metal peaks at 640 nm, 794 nm, and 974 nm, respectively, corresponding to ligand field transitions associated with the I5S→F55 transition of I49/2→F45/2 transition of ${\text{Nd}}^{3+}$ [29] and F27/2→F25/2 transition of ${\text{Yb}}^{3+}$ [55]. The extensive NIR absorption tail observed for NGQDs and RGQDs as well as sharp-featured NIR absorption peaks of Ho-NGQDs, Nd-NGQDs, and Yb-NGQDs enable their fluorescence excitation in the NIR-I and NIR-II biological windows with lowered absorption and tissue scattering of the excitation light. Since NIR fluorescence microscopy is limited to excitation with particular laser lines, excitation wavelengths of 650 nm, 808 nm, and 980 nm were chosen for Ho-NGQDs, Nd-NGQDs, and Yb-NGQDs at their respective absorption features. Not as restricted by their absorption spectra, NGQDs and RGQDs were excited at 808 nm. This wavelength located substantially deep within the NIR-I window for diminished tissue scattering and absorption can be generated by low-cost communication lasers democratizing this imaging approach.

**Figure 3.**
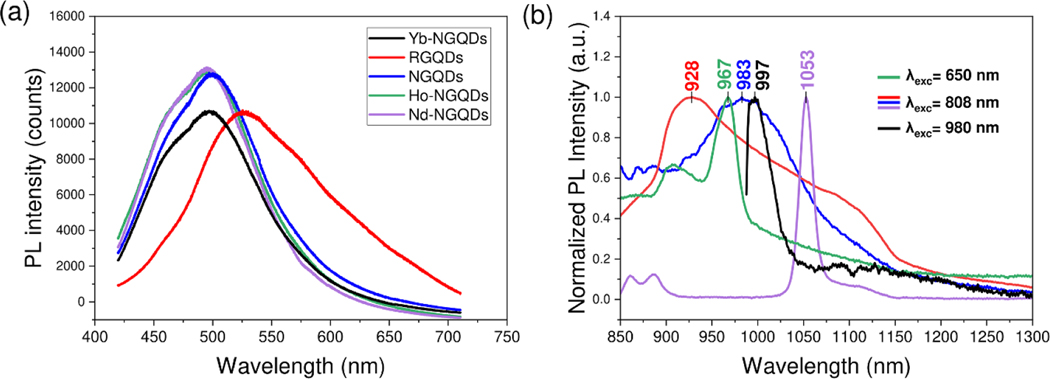
Normalized fluorescence emission of Yb-NGQDs (black), Nd-NGQDs (purple), NGQDs (blue), Ho-NGQDs (green), and RGQDs (red) in the visible with 400 nm excitation (a) and NIR (b).

All GQDs exhibit visible fluorescence attributed to the electronic quantum confinement within the regions of graphitic carbon on their graphitic surface (figure 3(a)) [56]. NGQDs, Nd-NGQDs, Ho-NGQDs, Yb-NGQDs, synthesized through the same ‘bottom-up’ method, possess similar broad fluorescence features peaking at ∼500 nm with 400 nm excitation, while the peak for RGQDs is shifted to 530 nm. While being less relevant for the present work, this visible fluorescence demonstrates the similarity in electronic configuration for all structures with slight changes produced by nitrogen doping and minimal to no influence from rare earth metals. The NIR fluorescence spectra of all the GQDs are, on the other hand, substantially different and appear to be dopant-dependent (figure 3(b)). Excited at 808 nm both RGQDs and NGQDs exhibit broad NIR emission features with peaks at 928 and 983 nm, respectively. A red shift of NGQD fluorescence can be dictated by surface passivation with nitrogen dopants. Nd-NGQDs excited at 808 nm exhibit fluorescence emission at 895 nm and 1053 nm corresponding to the F43/2→I49/2 and F43/2→I411/2 ligand field transitions of ${\text{Nd}}^{3+}$, respectively, with the most pronounced peak at 1053 nm [57, 58]. Thus, RGQDs, NGQDs, and Nd-NGQDs, excited with the same 808 nm laser can be used for imaging of multiple targets simultaneously via monitoring different NIR emission wavelengths (928 nm, 983 nm, and 1053 nm). Two fluorescence features at 906 nm and 967 nm are observed for Ho-NGQDs excited at 650 nm and attributed to the I58→I58 and I48→I56 transitions of ${\text{Ho}}^{3+}$ respectively [59, 60]. $\text{Ho}{\left({\text{NO}}_{3}\right)}_{3}\cdot 5{\text{H}}_{2}\text{O}$ does not exhibit NIR fluorescence on its own, which suggests that the GQDs platform protects ${\text{Ho}}^{3+}$ from strong water-assisted quenching. With 980 nm excitation Yb-NGQDs demonstrate a broader feature in the 990–1100 nm region arising from the crystal field splitting of the ${\text{Yb}}^{3+2}{\text{F}}_{7/2}$ ground state [61, 62]. Upon excitation at 400 nm, Yb-NGQDs produce similar fluorescence within 900–1100 nm, and a peak at 975 nm, corresponding to the F25/2→F27/2 transition of ${\text{Yb}}^{3+}$ (figure S4). Since 400 nm is not suitable for *in vivo* bioimaging applications, 980 nm excitation was further used. GQDs doped with Er also synthesized in this work, did not show any substantial NIR fluorescence with 808 nm or 980 nm excitation likely due to water quenching [63]. This results in having five spectrally-separated NIR GQD fluorophores (figure 3(b)).

**Figure 4.**
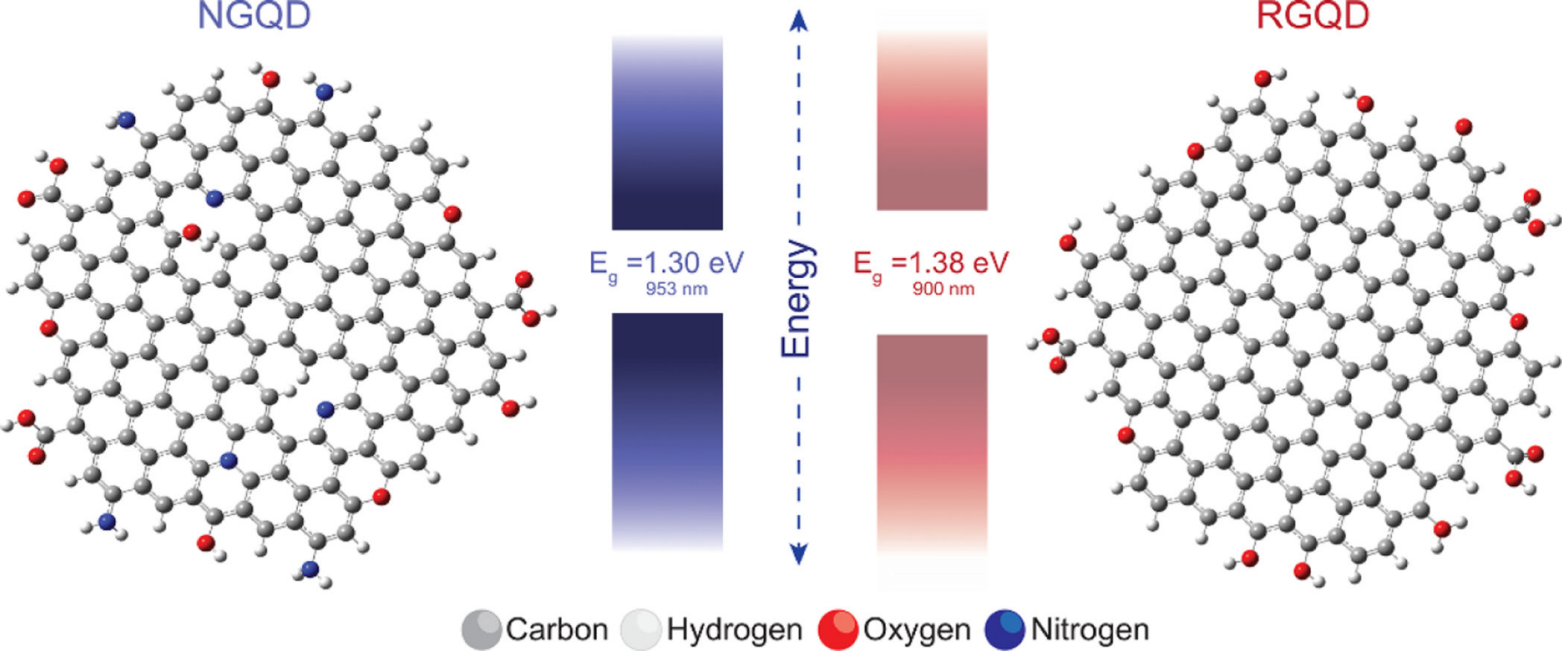
Computed ground state atomic structures of NGQD (C_145_H_35_O_17_N_7_) and RGQD (C_150_H_27_O_16_) with their NIR energy gaps in both eV and nm.

In order to describe the near-infrared fluorescence properties of NGQD and RGQD platforms we developed NGQD $\left({\text{C}}_{145}{\text{H}}_{35}{\text{O}}_{17}{\text{N}}_{7}\right)$ and RGQD $\left({\text{C}}_{150}{\text{H}}_{27}{\text{O}}_{16}\right)$ monolayer model systems computationally optimized in Gaussian16 program (figure 4). Monolayer models were employed in the interest of computational workload and it proved to be a sufficient estimation for this evaluation. Electronic properties, which were obtained via Hartree-Fock calculations, revealed NIR energy gaps at Eg=1.30eV for NGQD and Eg=1.30eV for RGQD (figure 4). The tighter gap of the NGQD, compared to the RGQD, suggests that the additional amine and nitro groups present in its structure can create mid-gap defect states. Corresponding computed fluorescence peaks of 953 nm for NGQD and 900 nm for RGQD (figure 4) monolayers were found to be slightly blue-shifted compared to experimental findings of 983 nm and 928 nm, respectively (figure 3(b)). This blue shift can occur because our single layer model structures have a more pronounced quantum confinement compared to the experimental multi-layered GQDs, as it was previously shown with layered structures of molybdenum disulfide [64]. These results demonstrate the possibility of NIR transitions for the proposed NGQD and RGQD structures, confirming our experimental findings.

While being an important characteristic of fluorophore’s NIR emission capabilities, NIR QY is usually very low and rarely assessed for nanomaterials. In order to fully characterize GQD biomarkers developed in this work, we utilized a comparative QY measurement with commonly used ICG dye as a reference. This showed QYs generally below 1% ranging between 0.09 and 0.69% (table 1). The QY of RGQDs was previously measured with the use of an integrating sphere as 6.29 ± 0.50% and 1.34 ± 0.15% with 637 and 808 nm excitation, respectively [26]. While still being on a lower end, QY exhibited by the GQDs is higher than that of a number of molecular NIR probes [36]. Furthermore, lower QYs can be compensated for by high photostabilities allowing for longer integration times and prolonged analyte monitoring. All GQDs developed in this work have demonstrated outstanding NIR fluorescence photostabilities in water within 4 h of laser irradiation compared to ICG dye, which quenched within 30 min of exposure (figure S5). The calculated signal to noise ratio for all GQDs after 4 h of laser irradiation was relatively high and exceeds that of the ICG after 45 min of laser exposure (table S1). These properties suggest that five spectrally separated GQD fluorophores developed in this work (NGQDs, RGQDs, Nd-NGQDs, Ho-NGQDs, and Yb-NGQDs) have a potential for simultaneous multiwavelength imaging and analyte detection. However, the applicability of these nanomaterials in biotechnology is strongly dependent on their biocompatibility.

**Figure 5.**
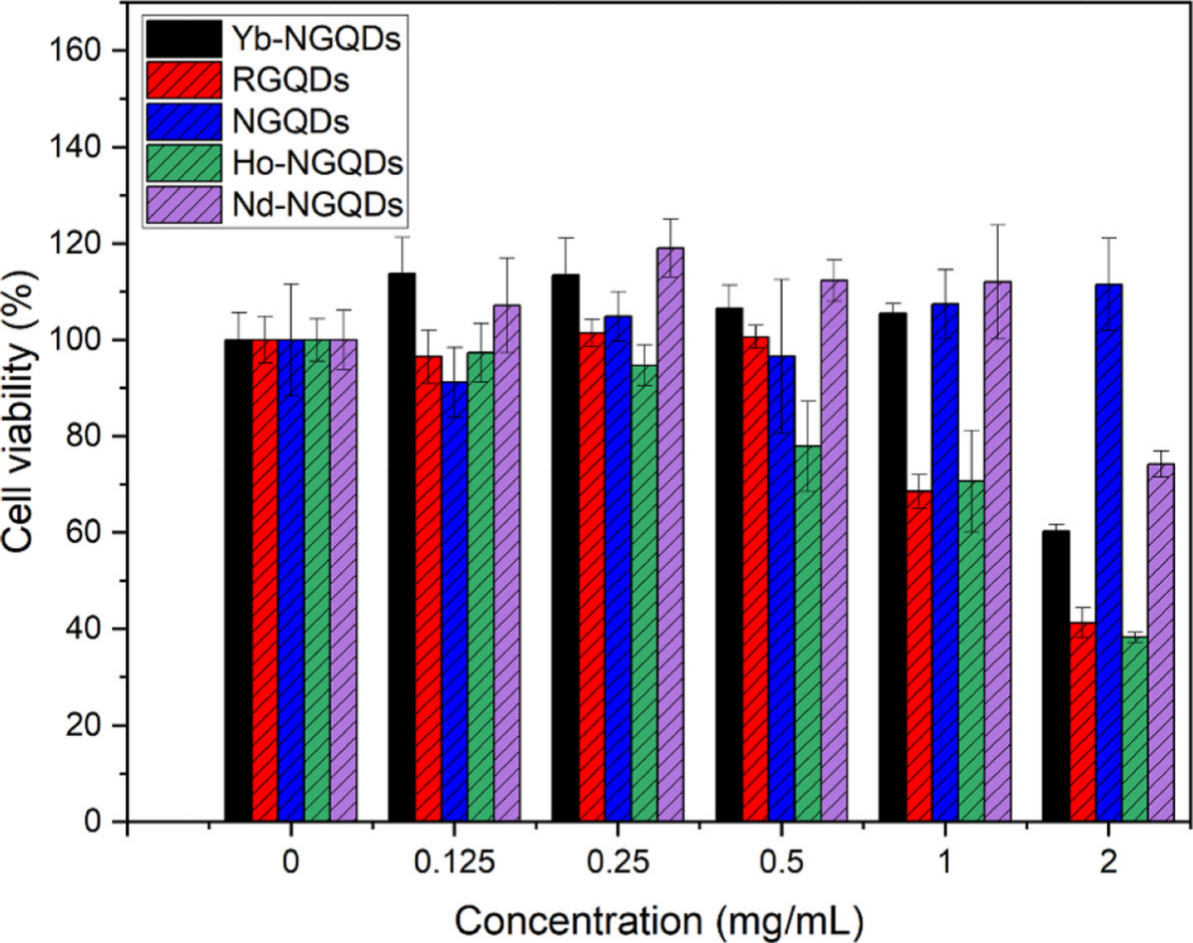
MTT assay viability of HEK-293 cells treated with Yb-NGQDs (black), Nd-NGQDs (purple), NGQDs (blue), Ho-NGQDs (green), and RGQDs (red).

The cytotoxicity of all GQDs was assessed in HEK-293 cells by the MTT assay (figure 5). Nanomaterials are considered generally biocompatible at above 80% cell viability which was achieved at 0.5 mg ml^−1^ for RGQDs and Ho-NGQDs, at 1 mg ml^−1^ for Yb-NGQDs and for Nd-NGQDs and at 2 mg ml^−1^ for NGQDs. This indicates that very low metal doping levels (<2.4%) do not drastically affect the biocompatibility of the new doped structures. The slight increase in NGQD cell viability has been previously related to the cellular metabolism of glucosamine precursor remains [28]. Moreover, all these GQDs have higher biocompatibility than some common NIR dyes, REM nanoparticles and SWCNTs [65–67]. For convenience, biocompatible concentrations of 0.5 mg ml^−1^ for RGQDs and Ho-NGQDs, and 1 mg ml^−1^ for NGQDs, Nd-NGQDs, and Yb-NGQDs, were chosen for further internalization/excretion studies in the HEK-293 cells.

In order to track GQDs inside the cells and assess the optimal timeline for imaging, a NIR fluorescence-based internalization/excretion study was performed *in vitro* via hyperspectral NIR fluorescence microscopy. Based on the GQD optical properties, NIR fluorescence emission within the cells was recorded with the following excitation lasers and only at specific emission wavelengths (table 2). Hyperspectral imaging allowed selecting a single wavelength specific to each GQD type and, thus, collecting its signal separately from potential autofluorescence and/or other dyes.

**Figure 6.**
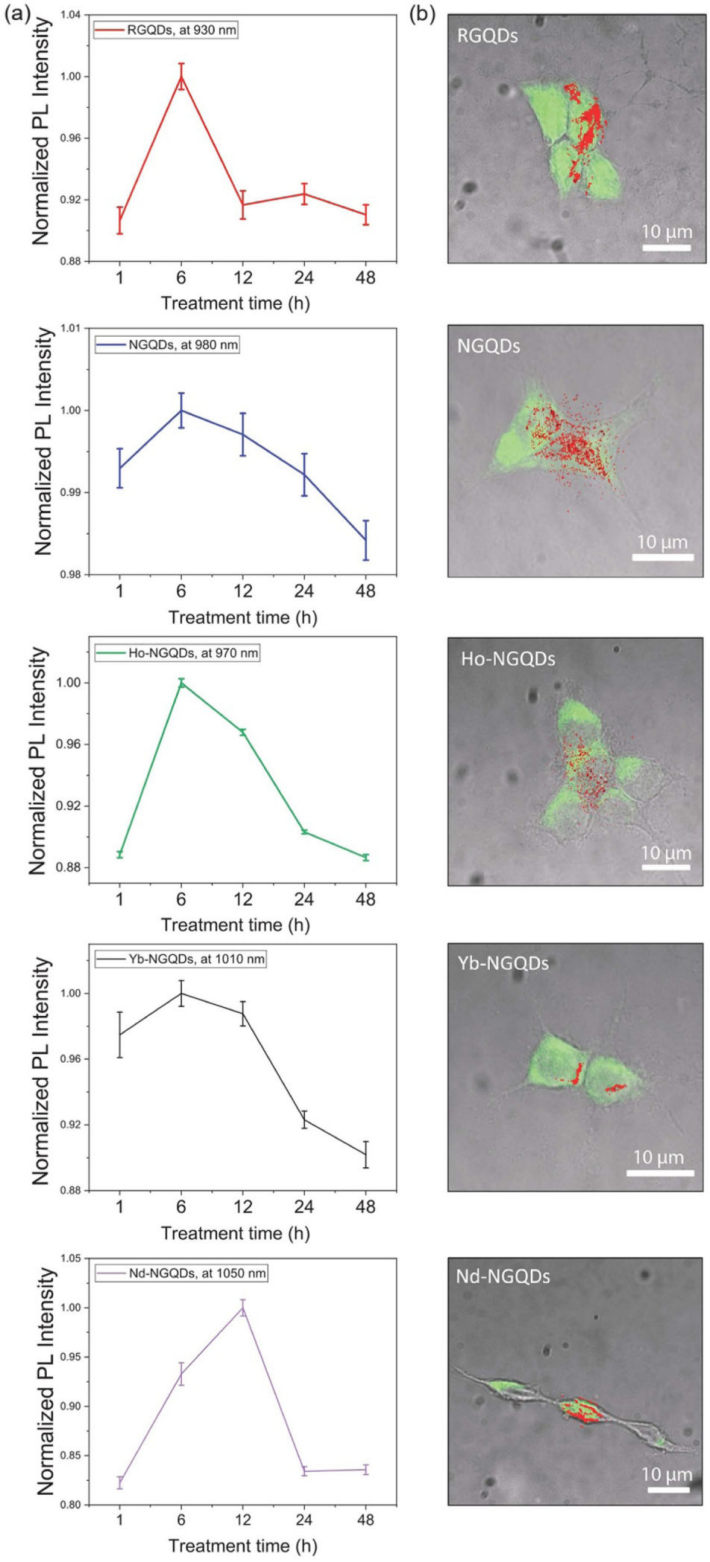
(a) Intracellular concentration of GQDs in HEK-293 cells over the period of 1–48 h analyzed by considering their average fluorescence intensity per unit cell area collected at their maximum emission wavelengths. (b) Bright-field/fluorescence overlay with visible and NIR GQD fluorescence collected with confocal visible and hyperspectral near-infrared microscopy. GQD visible fluorescence (shown in green) is excited with 460 ± 20 nm and collected using 535 ± 20 nm filters. Near-infrared GQD fluorescence (shown in red) is excited and collected at the wavelength specified in table 2 for each GQD type.

Each GQD suspension was introduced into the HEK-293 cells at its corresponding biocompatible concentration and incubated for 1, 6, 12, 24, and 48 h to provide a range of imaging timepoints tracing internalization. As GQDs internalized into cells, their intracellular fluorescence intensity normalized per unit cell area was utilized as a measure of internalization/excretion dynamics of the nanomaterial. All GQDs enter the cells with maximum accumulation at 6–12 h of incubation followed by excretion/degradation after 24 h (figures 6(a) and (b)). While confocal visible microscopy was utilized to ensure successful intracellular translocation of the nanomaterials, NIR hyperspectral imaging (figure 6(a)) demonstrated their capacity for NIR image tracking within the cell. Since RGQDs and Nd-NGQDs have higher NIR QY than other GQDs, their imaging required shorter integration times (figure S6). Minimal to no emission was detected in the extracellular environment as GQDs were removed by a preliminary washing step leaving only those that internalized. It is also apparent that all the GQDs exhibit bright intracellular fluorescence in the visible and in the near-infrared (figures 6(b) and S6) suggesting that all five synthesized structures can serve as visible and, most importantly, NIR fluorescence markers.

**Figure 7.**
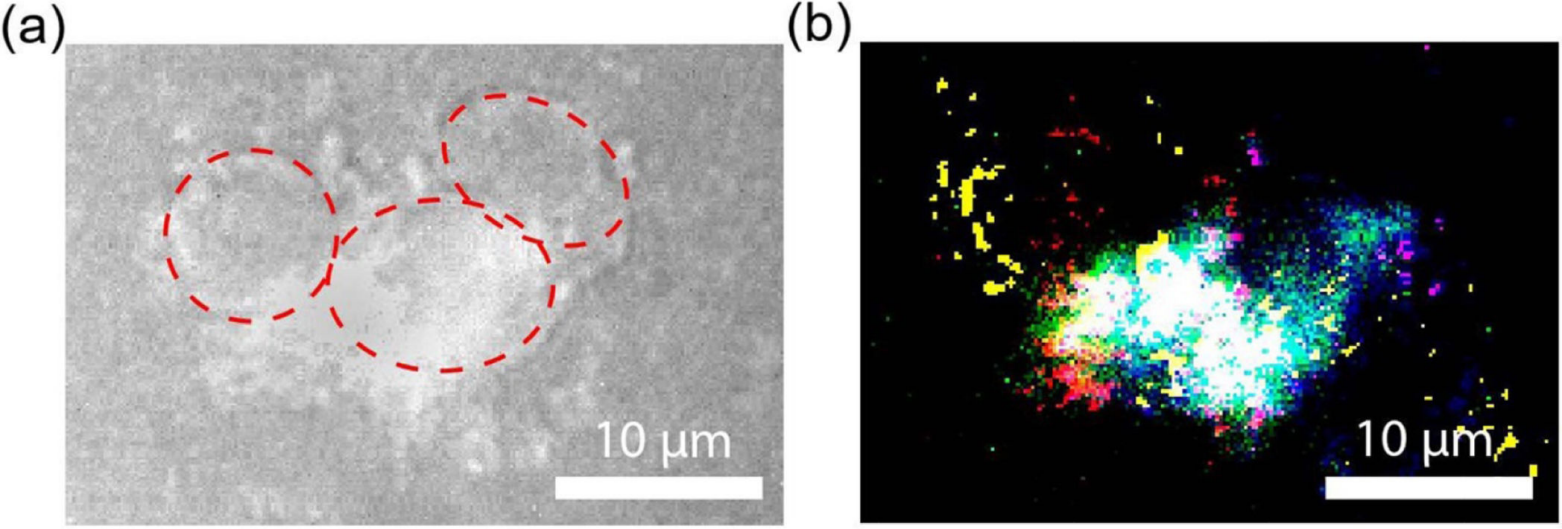
(a) Broadband NIR image (900–1600 nm) of the HEK-293 cells incubated with all five GQDs. The cells are outlined based on the bright field image (figure S8). (b) A false-colour fluorescence image of the same region colored by the type of GQDs (RGQDs (red), Ho-NGQDs (green), NGQDs (blue), Yb-NGQDs (yellow), and Nd-NGQDs (magenta)).

Their capability for multianalyte detection and multiplex imaging was further verified by introducing all five GQD types into the same well with HEK-293 cells and imaging them all together at a 6 h time point. In this experiment 650 nm, 808 nm and 980 nm lasers were utilized one-by-one to excite different types of GQDs, while their fluorescence was recorded using the hyperspectral imager at specific emission wavelengths of their respective fluorescence maxima (table 2). Overlaid fluorescence signals showed successful co-internalization of all the GQDs with substantial signal overlap (figure 7). Looking at each wavelength channel separately (figure S7), it is possible to spectrally distinguish the location of each of the GQD type within the cells. This verifies the possibility of multiplex imaging with up to five different GQDs, which can be used for simultaneous tracing of five different therapeutic agents in combination therapies or sensing up to five different analytes *in vitro.* Furthermore, considering high tissue penetration depth at all the NIR excitation and emission wavelengths used in this work, these GQDs have a significant potential for multiplex imaging *in vivo*.

## 4. Conclusion

In this work, we have for the first time developed and tested the feasibility of five novel biocompatible NIR imaging platforms: RGQDs, NGQDs, Nd-NGQDs, Yb-NGQDs, and Ho-NGQDs. These GQDs were synthesized using cost-effective ‘top-down’ and ‘bottom-up’ synthetic procedures resulting in 4–11 nm graphitic nanostructures with either oxygen or oxygen and nitrogen-containing functional groups. Nd-NGQDs, Yb-NGQDs, and Ho-NGQDs also contain few percent of corresponding rare earth metal dopants. Synthesized nanostructures appear to be highly biocompatible at up to 0.5–2 mg ml^−1^ concentrations, allowing the use of substantial GQD doses for high contrast imaging. With 650 nm, 808 nm, and 980 nm laser excitation, Ho-NGQDs, NGQDs, RGQDs, Nd-NGQDs and Yb-NGQDs exhibit fluorescence emission within NIR-I and NIR-II biological windows with peaks at ∼970 nm, 980 nm, 930 nm, 1050 nm and 1010 nm, and NIR QYs of up to 1.34%. Their NIR fluorescence is remarkably photostable within 4 h of continuous irradiation as compared to that of conventional fluorophores. This allows for long signal accumulation times compensating for lower NIR QYs. The capability of *in vitro* fluorescence tracking has been evaluated for all five GQD types in HEK-293 cells with all showing successful internalization maximized at 6–12 h and effective intracellular imaging in the visible and NIR. Furthermore, the ultimate ability of using five GQDs with five different emission wavelengths for multiplex imaging in the NIR has been verified for the first time in this work in HEK-293 cells. While such capabilities are well-developed for visible fluorescence tracking, this work introducing five spectrally-separated NIR fluorophores provides a critical advancement for NIR imaging, where multiplexing was rarely an option. These novel nanomaterials can address the critical need for simultaneous bioimaging and further enable real-time multidrug delivery and multianalyte detection *in vivo* via high penetration depth NIR fluorescence.

The major potential clinical impact of this work includes the capability of imaging multiple low-depth targets. For instance, this may enable simultaneous tracking of several combination therapies delivered by different types of NIR GQDs to clinical targets within several centimeters under the skin including low-lying tumors as well as targets in the brain. NIR imaging at different wavelengths can also aid in multianalyte detection within ‘lab on a chip’ implantable sensors. In those, NIR GQD fluorescence can provide non-invasive optical detection mechanism. Attaching probes for various analytes to different NIR GQD types will therefore allow for their simultaneous detection. These approaches may face such engineering challenges as limitations for the depth of imaging applications down to few centimeters and cost limitations for NIR detectors. In order to avoid those, GQDs, fluorescing at the wavelengths of up to 1000 nm limit of silicon chip sensors, may be preferentially selected.

## Data availability statement

All data that support the findings of this study are included within the article (and any supplementary files).

## Supplementary Material

Supplementary Materials

## Figures and Tables

**Table 1.** Comparative measurements of QY of GQDs and ICG standard.

Sample	Solvent	Excitation	Quantum Yield (%)

Nd-NGQDs	Water	808 nm	0.68
NGQDs	Water	808 nm	0.22
RGQDs	Water	808 nm	1.34 [26]
Ho-NGQDs	Water	650 nm	0.09
ICG	Water	808 nm	2.5 [39]

**Table 2.** Excitation and emission parameters used for internalization/excretion study of GQDs in HEK-293 cells.

Sample	RGQDs	Ho-NGQDs	NGQDs	Yb-NGQDs	Nd-NGQDs

Excitation (nm)	808	650	808	980	808
Emission (nm)	930	970	980	1010	1050
