# Supplementary material for: Five near-infrared-emissive graphene quantum dots for multiplex bioimaging
Source: 2d Mater. Author manuscript; Available in PMC 2024 Aug 15. (PMC11326670; doi:10.1088/2053-1583/ad1c6e)
Supplement: Supplementary Materials [file NIHMS1964389-supplement-Supplementary_Materials.pdf]

Five Near-Infrared-Emissive Graphene Quantum Dots for Multiplex Bioimaging.

*Alina R. Valimukhametova, Olivia Fannon, Ugur C. Topkiran, Abby Dorsky, Olivia Sottile, Roberto Gonzalez-Rodriguez, Jeffery Coffer, Anton V. Naumov**

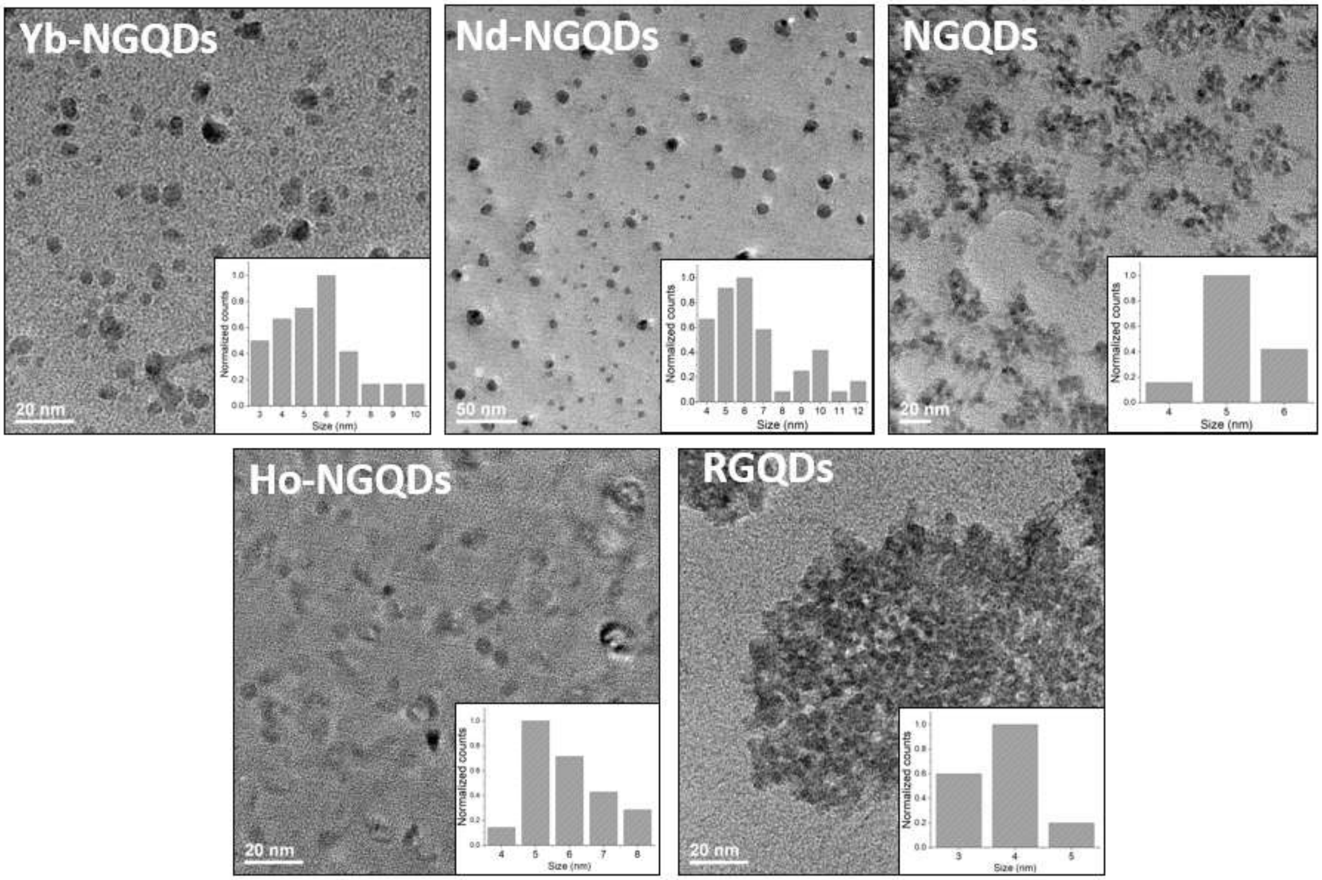

Figure S1. TEM images and size distributions of NGQDs (5.7±1.0 nm), Nd-NGQDs (5.5±1.1 nm and 11.0±1.6 nm), Ho-NGQDs (5.9±1.2 nm), Yb-NGQDs (5.6±1.8 nm), and RGQDs (4.0±0.7 nm). For Nd-NGQDs the average of two peaks was chosen to provide a mean particle size.

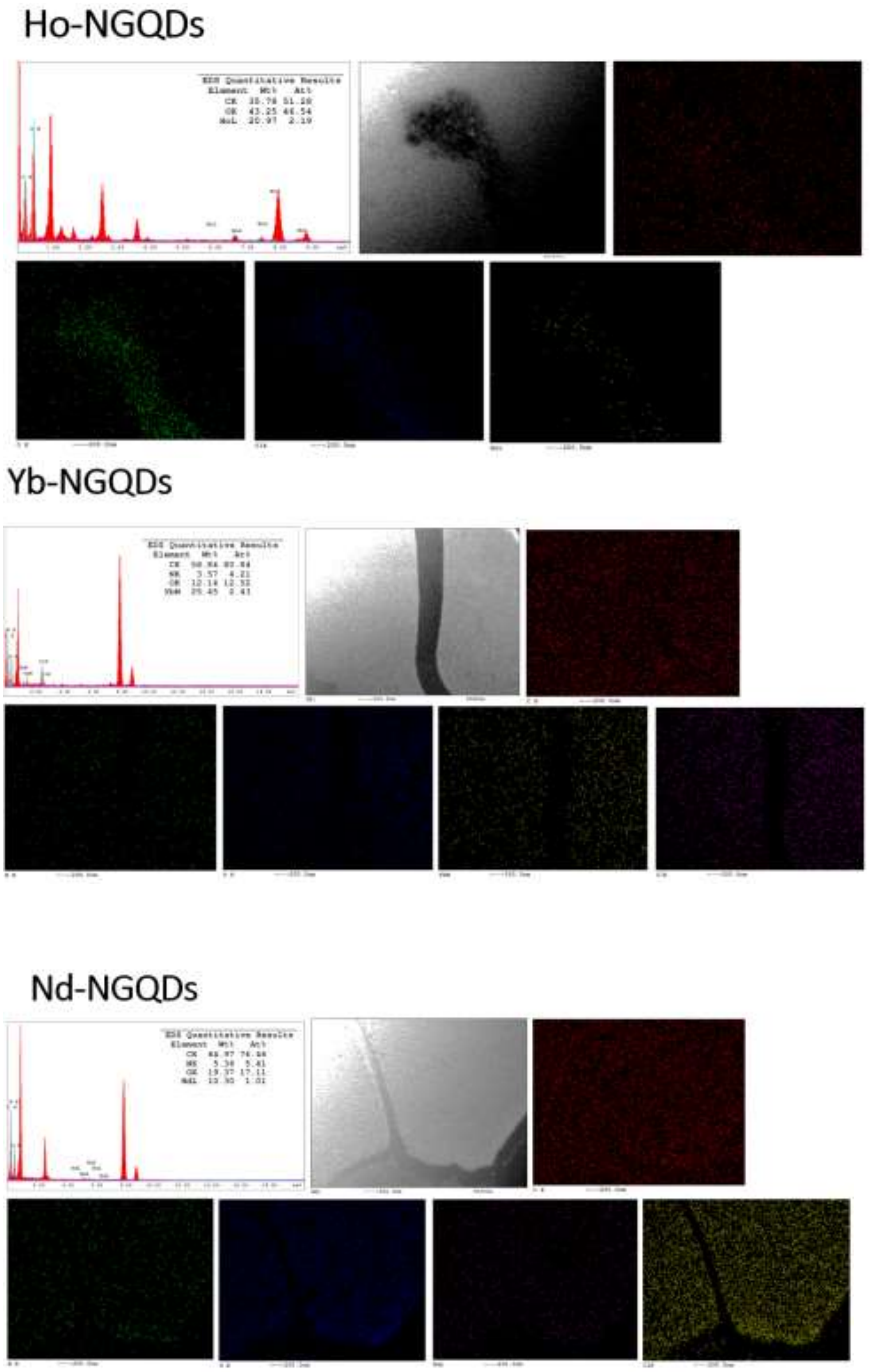

Figure S2. EDX spectra, weight and atomic percentages, TEM images and EDX elemental maps (by element) of the selected region containing Ho-NGQDs, Yb-NGQDs, and Nd-NGQDs.

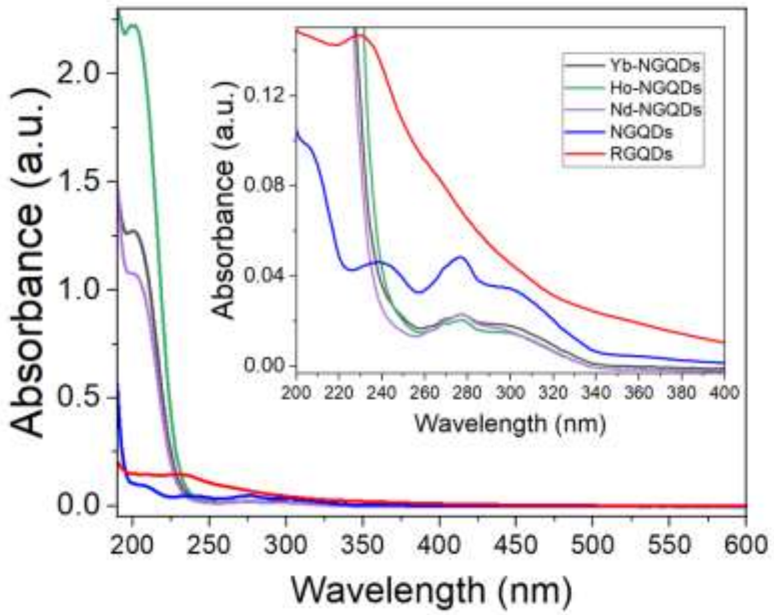

Figure S3. UV-VIS absorption spectra of Yb-NGQDs (black), Nd-NGQDs (purple), NGQDs (blue), Ho-NGQDs (green), and RGQDs (red) within 190 – 600 nm range. Inset: zoom in on the 200 – 400 nm range.

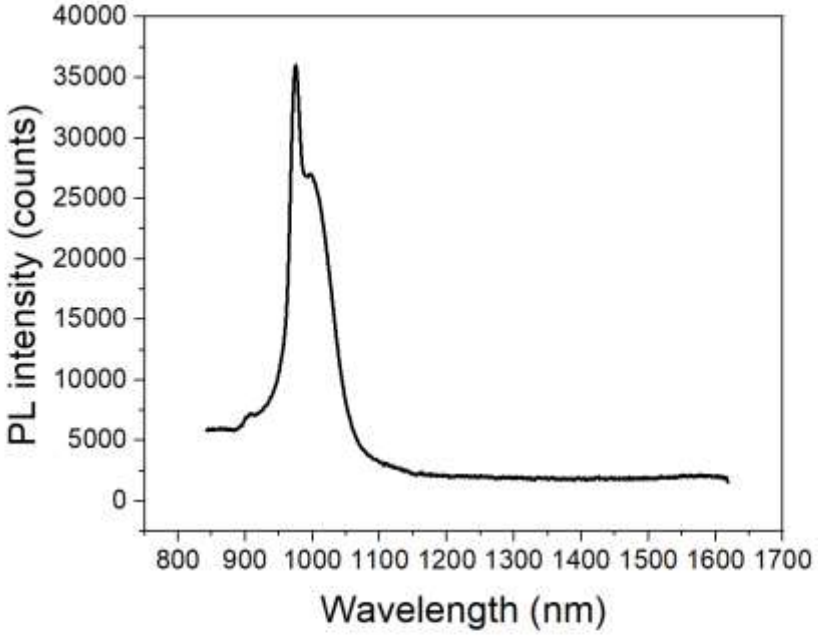

Figure S4. Fluorescence emission of Yb-NGQDs with 400 nm laser excitation.

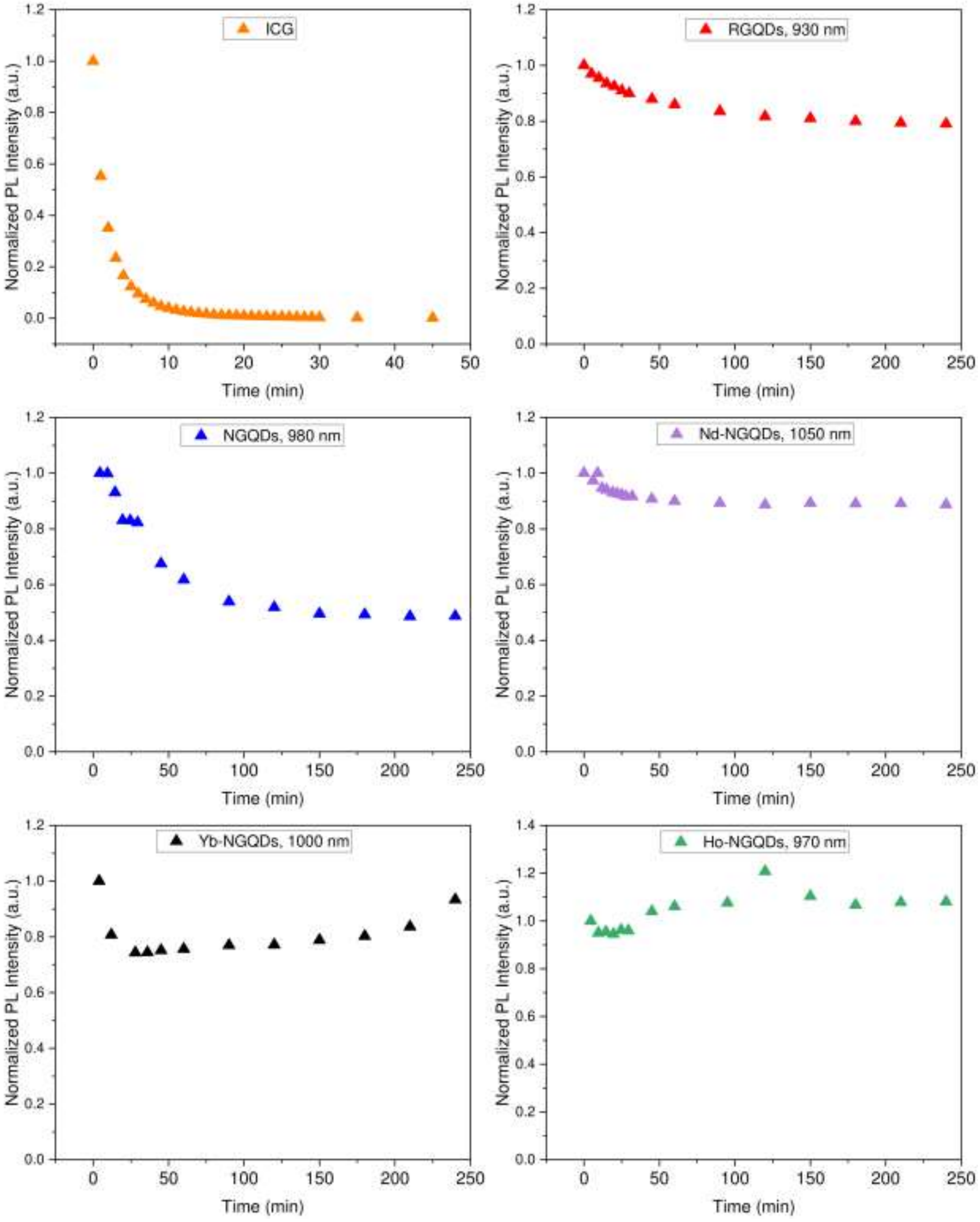

Figure S5. Photostability of ICG, RGQDs, NGQDs, and Nd-NGQDs under 808 nm laser exposure, Yb-NGQDs under 980 nm laser exposure, Ho-NGQDs under 650 nm laser exposure at a power density of 0.2 W cm^{-2}.

Table S1. Signal to noise ratio (S/N) calculated for all GQDs after 4 h and for ICG after 45 min of laser irradiation through the First Standard Deviation (FSD) method using the following equation

$$\frac{S}{N} = \frac{S_{peak} - S_{background}}{\sqrt{S_{background}}}$$

	RGQDs	Nd-NGQDs	Yb-NGQDs	Ho-NGQDs	NGQDs	ICG
S/N	1473	395	112	49	23	7

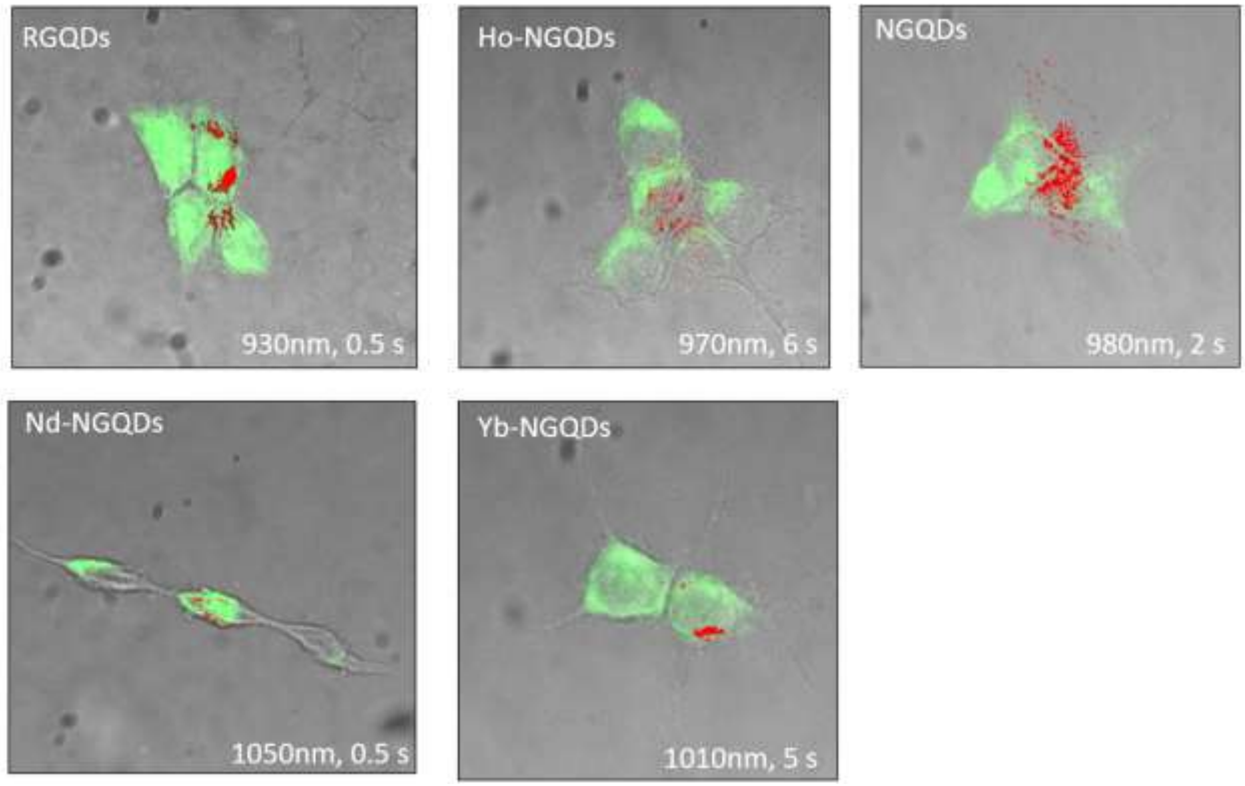

Figure S6. Bright-field/NIR fluorescence / VIS confocal fluorescence overlay images of HEK-293 cells treated with Yb-NGQDs, Nd-NGQDs, NGQDs, Ho-NGQDs, and RGQDs for 12 h.

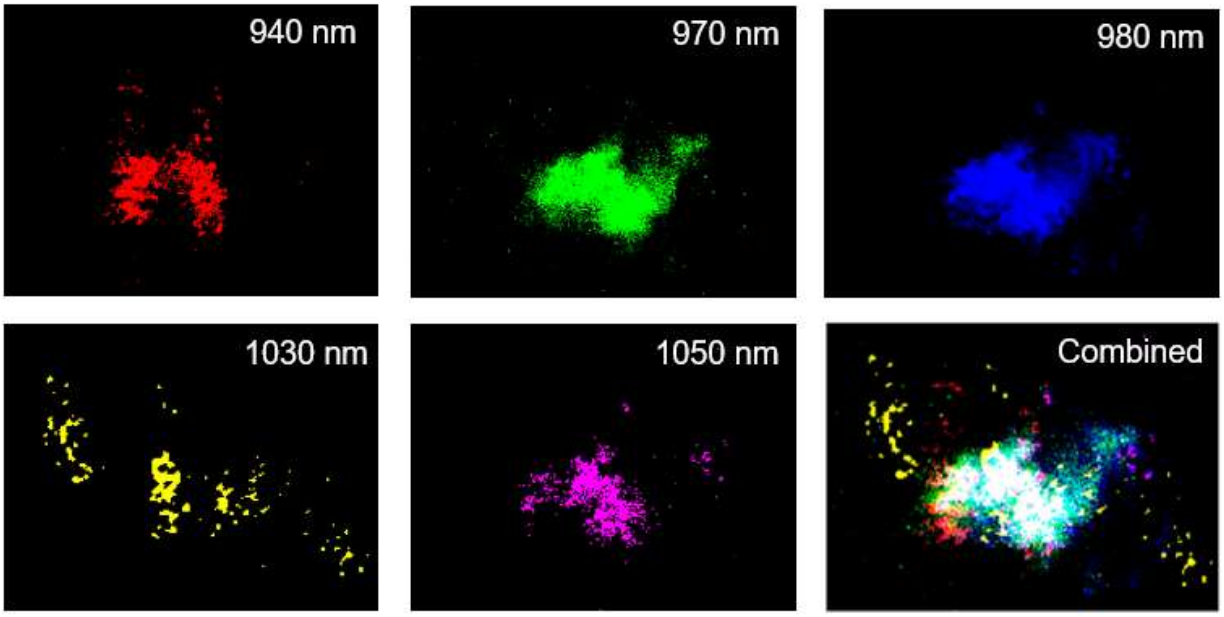

Figure S7. A false-colour fluorescence images of five types GQDs internalized into of HEK-293 cells colored by the type of GQDs (RGQDs (red), Ho-NGQDs (green), NGQDs (blue), Yb-NGQDs (yellow), and Nd-NGQDs (magenta)), taken at each wavelength channel separately and combined together.

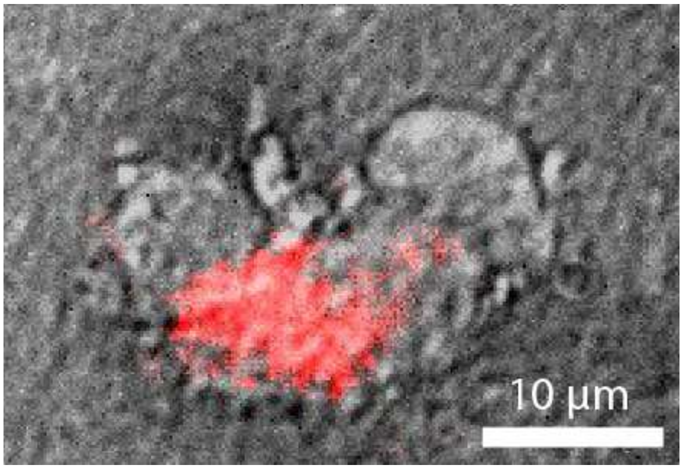

Figure S8. Bright-field/NIR fluorescence overlay image of HEK-293 cells taken with an InGaAs NIR camera. Near-infrared GQD fluorescence is shown in red (900-1600 nm emission).
